# A Ship’s Log

**Published:** 1881-09

**Authors:** 


### A SHIP'S LOG.

The speed of vessels is approximately determined by the use of the log and log-line. The log is a triangular or quadrangular piece of wood about a quarter of an inch thick, so balanced by means of a plate of lead as to swim perpendicularly in the water, with about two-thirds of it under the water. The log-line is a small cord, one end of which, divided into three, so that the wood hangs from the cord as a scale-pan from a balance-beam, is fastened to the log, while the other is wound round a reel in the ship. The log, thus poised, keeps its place in the water, while the line is unwound from the reel as the ship moves through the water, and the length of line unwound in a given time gives the rate of the ship's sailing. This is calculated by knots made on the line at certain distances, while the time is measured by a sand glass of a certain number of seconds. The length between the knots is so proportioned to the time of the glass that the knots unwound while the glass runs down show the number of miles the ship is sailing per hour. The first knot is placed about five fathoms from the log, to allow the latter to get clear of the ship before the reckoning commences. This is called the stray-line.

"THE only lady that ever impressed me much," said an old bachelor, "was a 300-pound woman, who was standing in a car, and when the car turned a corner fell against me."

